# Surgery

**Published:** 1857-01

**Authors:** 


### SURGERY.

1. LACERATION OF THE PERINEUM. CASE SUCCESSFULLY OPERATED ON BY ISAAC BAKER BROWN'S METHOD. By S. W. Butler, M.D., of Burlington, N. J.—[The October No. of the New Jersey Medical and Surgical Reporter contains an interesting account of a case of lacerated perineum operated on successfully by the editor after the method of Mr. Brown, of London. The history of the case and operation is preceded by a valuable synopsis of the literature of the subject, but our space will permit the introduction of only the former.]

"In June, 1856, I was consulted by Mrs. D——, æt. 38, who was suffering much in health and comfort, in consequence of a rupture of the perineum which occurred at the time of her seventh confinement, April 2d, 1853, more than three years since. The child was born suddenly by one powerful expulsive pain, before the arrival of her medical attendant. Her general health was failing, and her condition rendered her a source of discomfort to herself, and of annoyance to those about her. She was troubled with both uterine and vesical prolapse, the latter condition so interfering with the functions of the bladder as to render that organ and its secretion a source of great irritation to the system, amounting at times to severe strangury. In addition, she had scarcely any control over the secretion from the bowels. Especially was this the case in warm weather, when she sometimes found it necessary to sit for days and nights together on a night-chair. Wherever she was, too, and at all times, she and those around her were subject to the constant annoyance of intestinal gases, over the escape of which she had not the slightest control. In her first confinement, Mrs. D. had a slight laceration of the perineum, though it gave no special difficulty, and she never informed her medical attendant of it. Although naturally a woman of weak nerves and rather slender constitution, rendered much worse, of course, by having been for so long a time in such a deplorable condition, finding that she was growing worse, that life had become burdensome in her present condition, and fearing to encounter the heat of another summer, she resolved to have an operation performed, in hope of obtaining relief. An examination of the parts revealed the fact that there was complete rupture of the perineum, involving the sphincter ani, and a limited portion of the recto-vaginal septum. The hope and belief were held out to her that she could be relieved, and perhaps entirely cured, by an operation, while the fact was not concealed that her health had suffered much, and that her present condition in this respect, and the ordinary dangers attending every severe operation, would have to be encountered. She was also forewarned as to the severe ordeal she would have to pass through in the treatment succeeding the operation. She bravely, however, and, as the result proved, fortunately for her, resolved to encounter all and submit to it.

"On the 9th of June, 1856, at 4 P.M., her bowels having been cleared out by a dose of medicine and an injection, and having passed water a short time previously, with the assistance of Drs. Gauntt and Pugh, I proceeded to operate. She was placed on a table in the position for lithotomy, and the hair about the parts closely shaved off. An anæsthetic was used, consisting of ether three parts, and chloroform one part. When she was sufficiently under the anæsthetic influence, the sides of the fissure, which we found somewhat uneven and rough, were pared so as to present clean, raw surfaces to each other, without the interference of either skin or mucous membrane, to prevent perfect union. This part of the operation was considered very important, and was much more tedious than we had supposed it would be. The next step was *to divide the sphincter ani* on both sides of the os coccygis, about a quarter of an inch in front of its attachment to that bone, by an incision carried outwards and backwards. These incisions were about an inch in depth, and *should have been deeper*, as we found that we were somewhat embarrassed in our after-treatment by the action of the sphincter. *This is an important part of Mr. Brown's operation, the object being to destroy muscular traction, for so long as it exists it interferes with the union of the parts.* The next step was the insertion of the quill sutures, pieces of a gum elastic bougie being used for quills, and stout linen ("patent") thread, well waxed, being used for sutures. Being disappointed by the instrument-maker in procuring the form of needle recommended by Mr. Brown, a Physic's forceps was armed with an ordinary crooked needle, with which, and with a long crooked needle with a straight shaft, we succeeded with little inconvenience in introducing three sutures, taking care to have them enter the tissues an inch external to the margin of the denuded surface, and carrying it deep through the tissues nearly to the bottom of the fissure, hard by the mucous membrane of the vagina and rectum. The suture next the rectum was inserted first. The three sutures having been inserted, the next step was to place dossils of oiled lint into the incisions into the sphincter ani, to prevent the wound healing by the first intention, thus interfering with the process of healing between the pared surfaces, by muscular traction. The quills were then introduced, and the threads drawn moderately tight over them and tied. This done, two interrupted sutures were introduced for the purpose of bringing the skin into perfect coaptation. We supposed at the time that two sutures would be sufficient, but afterwards wished we had followed Mr. Brown's suggestion, and inserted three or four, as the union of the edges of the wound would have been more speedy and perfect. The parts were then examined by inserting one finger into the vagina, and the other into the rectum, and found to be in the desired condition. After thoroughly cleansing the parts, a dry lint dressing was applied for the present, secured by a T bandage, and the patient transferred to the bed, lying on her left side, with the thighs flexed on the abdomen, and the legs on the thighs, the knees being bound together by a few turns of a linen roller. There being a desire to pass water, introduced, the catheter, and drew off a pint of limpid urine. Gave two grains of opium and one of camphor.

"The operation lasted a little over an hour, and was well borne by the patient. During the most painful part of the operation, and until near its close, patient was so completely under the anæsthetic influence of the chloric ether, that she was entirely unconscious of pain. Indeed, "imagination winged her fancy," so that while undergoing the operation, she was travelling in the States of Illinois and Missouri, where she had resided several years. She said she never was so happy. The ether, however, gave rise to a restlessness that interfered somewhat with the operation, a disadvantage which, according to our observation, does not attend the anæsthetic influence of chloroform when used alone."

[We omit the subsequent full details of the case. However, on the sixth day, everything having gone on well, the deep sutures were removed, and on the same day, in consequence of violent efforts to control the passage of flatus with which she had been troubled, and a rather imperfect division of the sphincter, the wound thus producing a fistulous communication, partly opened. On the ninth day the superficial sutures were removed. On the succeeding day fistulous openings were discovered at the verge of the anus, and at the posterior fourchette, which were touched with nitrate of silver. The wound had healed externally about an inch. There was a fissure at the verge of the anus. On the thirteenth day the recto-vaginal septum was examined, and *union appeared to be perfect throughout.* The fistulous opening was subsequently touched with tinct. cantharides, and on the nineteenth day had entirely healed. Two months after the operation it was found upon examination that there was about an inch of renewed perineum, and the parts perfectly sound and healthy, with the exception of a weakness of the sphincter ani, which prevented a perfect control of flatus. The recto-vaginal septum was perfect.]

2. CHRONIC ABSCESS OF THE LOWER END OF THE TIBIA.

"IN 1844 the leg was broken about three inches above the ankle joint; the fracture united kindly, but from that day until this he has suffered more or less pain in the limb. At length an opening occurred in the lower part of the leg, and pus was discharging. Dr. Hamilton amputated the leg near its middle, on the 4th of last month.

"The inflammation and consequent softening of the bone extends to about six inches above the joint, the whole of this portion being enlarged by an expansion of its structure, and by the conversion of its lamellated tissue into cellular, so that the structure of that portion of the diaphysis which is involved cannot be distinguished from the epiphysis.

"The cavity of the abscess is in the lower end of the bone, and is two inches in length in its long diameter, and one in its short. At two points the walls have been perforated by ulceration or absorption, forming cloacæ. The whole was lined with a perfectly organized and quite firm pyogenic membrane. The fibula has not partaken in the disease.

"It was believed that amputation would afford the only remedy, as the disease had existed so long a time, and the openings in the bone, which were free, and had existed also a long time, had failed to give any relief."—*Proceedings of Buffalo Med. Association.—Buffalo Med. Journal,* Nov. 1856.

3. MODE OF REDUCING DISLOCATION OF THE THUMB. By John Doe, M.D., of Cabot, Vermont.

"Having had occasion to reduce a dislocation of the thumb several times, when the first phalanx is thrown upon the dorsum of the metacarpal bone, and having never encountered any particular difficulty in effecting it, it has been a cause of surprise to me that the method I am now to describe is not more generally known. There is not an English writer on surgery, from Sir A. Cooper to Fergusson, nor an American one, so far as I know, that alludes to this method; and if we are to judge from an article by M. Demarquay, published in the Medical News, of May, 1852, and accredited to the *Bulletin de Thérapeutique*, it might well be presumed that the French are also innocent of practising or teaching it.

"The common method of reducing this luxation is, as is well known, to flex the thumb, fasten upon it a tape with a clove hitch, and with this make extension. If this, or more violent means, does not succeed, we are directed either to abandon the attempt at reduction, or what is still worse, effect it by making incision, or by amputation of the end of the metacarpal bone. Extension has sometimes been persevered in to such a degree that the soft parts have been lacerated, or the thumb actually torn off; and in Braithwaite's Retrospect, part xxii, M. Blandin describes a forceps well calculated to do this.

"In this dislocation, the phalangeal end of the metacarpus projects into the palmar surface of the hand, forcing itself between and through the flexor muscles of the thumb, which form a loop around the head of the bone. Extension made upon the thumb makes this loop more tense; and as the metacarpal end of the first phalanx is broad and considerably flattened on its palmar aspect, it must be apparent at once that the difficulty of reduction is directly as the amount of extension. There is good reason to believe that extension would never succeed in these cases without rotation. The principal indication in treatment here is to relax the flexor forming the loop, so that the end of the phalanx can be pushed forward into the loop, and by carrying the muscles forward with it, disengage the head of the metacarpus. This can be done in the following manner:

"Having previously warmed the hand, if cold, in warm water, the surgeon should seat himself by the patient, facing in the same direction, and upon the same side with the injured thumb, and place the hand upon his knee. Tip back the thumb upon the dorsum of the metacarpus to more than a right angle, or so as to form a slightly acute angle with the latter bone; place both index fingers against the ball of the thumb, and the ends of both thumbs against the dorsum of the disarticulated end of the phalanx; now by pushing forward forcibly, get steadily against the phalangeal bone with both thumbs. Reduction will generally be effected on the first trial, and almost instantly.

"The writer claims not the honor of originating this method, but supposes it to be adopted by many surgeons in this country."—*New Orleans Med. News*, Dec. 1856.

4. TREATMENT OF RANULA.—M. Gosselin, after alluding to the various modes of treating ranula that have been adopted, and the relapses that are so common after them, describes the plan that he has himself found beneficial. He first of all performs excision, as recommended by Boyer, and then cauterizes with the nitrate of silver. Next day he introduces a probe into the wound, owing to its tendency to close, and repeats the cauterization the day after that. On the third or fourth day he enlarges, by means of the scissors, the aperture, which has become too narrow, and on the following day cauterizes again. After ten or twelve days of this assiduous attention, if on the introduction of a probe he finds the cavity is obliterated, he leaves the opening to itself. If, however, a track of a certain extent still exists, he again enlarges the orifice with scissors. This attention to the case is rarely required beyond fifteen days, when the external opening becomes closed, and the cavity being obliterated, there is no fear of relapse. M. Gosselin has operated in this way in several cases, and in three of these, which he has watched for several years, no relapse has ensued, the opening remaining closed. This plan of procedure has also been extended to various analogous cases, in which there is a cavity with secreting walls, having no spontaneous tendency to approach each other.—*L'Union Médicale.—Boston Med. and Surg. Journal*, December 4th, 1856.

5. BALSAM COPAIBA AS AN INJECTION IN GONORRHŒA.—Mr. Dallas, of Odessa, states, in confirmation of the observations already published by Taddei, Marchal, and others, that the injection of the balsam of copaiba is the most efficacious mode of treating gonorrhœa:—In sixteen cases he has so employed it, using no internal remedy, either in recent or old gonorrhœa, with complete success. His formula is copaib., five drachms; one yolk of egg; gummy extract of opium, one grain; water, seven ounces. The injection should be used several times a day.—*Gaz. des Hôpitaux.—Ibid.*

6. PERCHLORIDE OF IRON AS A HEMOSTATIC.—A correspondent of the "Moniteur des Hôpitaux" (1856, No. 24), states, that one of the principal elements of success in the difficult and dangerous operations M. Maissonneuve is famous for undertaking, is the remarkable use he makes of hemostatics during their performance. He cites a recent case, occurring in a lad of sixteen, of fungus tumor of the dura mater, the growth of which, after having been temporarily arrested by ligature of the carotid, took on enormous proportions, and was accompanied by exhausting hemorrhages. M. Maissonneuve determined upon its removal, but the tumor bled on the slightest contact, and the patient would not be able to bear the slightest loss of blood. The line of incision extended from the anterior parts of the ear to the summit of the head, and descending along the nose, was carried backwards, and then upwards to the base of the jaw, and its point of departure. A great number of arteries were thus divided, five or six of which, by reason of their anastomotic enlargements, had acquired almost the size of the radial artery. Intelligent assistants immediately compressed them with the finger, but it was impossible to thus continue the dissection without exposing the patient to the danger of death from syncope. M. Maissonneuve therefore applied to each vessel a little pledget of charpie, soaked in perchloride of iron, which was allowed to attach itself to the wound. At every stroke of the bistoury or scissors he applied a new plug, so that during the operation the patient scarcely lost a spoonful of blood; and when the tumor had been entirely removed, the entire surface of the wound was found completely dried and tanned, and was at once dressed, without the necessity of the application of a single ligature. The brown eschar which covered the wound was detached about the 20th day, without giving rise to any hemorrhage; and although the cure can scarcely be expected to prove radical, the patient for the present is perfectly well.—*Monthly Stethoscope*, Nov. 1856.

7. SURGICAL STATISTICS OF THE MASSACHUSETTS GENERAL HOSPITAL. By *S. Kneeland, Jr., M.D.*, of Boston.—During the preparation of an analytical index of the Surgical Records of the Massachusetts General Hospital, from its establishment in 1821 to 1856, a period of twenty-five years, some facts of statistical value came to my notice, which may prove interesting to the profession. I select a few of the most striking, alphabetically arranged, to obtain which it would be necessary for the student to employ several hours of tedious examination of manuscript.

AMPUTATIONS.—Of the *arm*, 20 cases, of which 4 were fatal, though only 2 depended on the operation itself; the other 2 were scrofulous cases.

Of the *fore-arm*, 18 cases; 1 death.

Of the *leg*, 90 cases, and 19 deaths, caused either by the severity of the accident, fracture of the skull, or other accompanying injury.

At the *shoulder-joint*, 6 cases, and only 1 death, although the accidents were very severe.

Of the *thigh*, 97 cases and 26 deaths, of which 5 were from the severity of the accident.

ARTERIES, *ligature of.*—The *external iliac* was tied 4 times, with 1 death in 2 days. The *internal iliac* was tied once, proving fatal in a week.

CANCER.—*Of the lower lip*, 58 cases, of which only 5 were in females, and generally beyond the age of 50. In most, the habit of pipe-smoking was acknowledged. Many eminent surgeons, among others the late Dr. J. C. Warren, have considered this habit one of the most frequent exciting causes of labial cancer.

*Cancer of the tongue*, 20 cases, of which 5 were females—almost always in tobacco-poisoned mouths. This comes on at an earlier age than cancer of the lip.

EPULIS, 9 cases, all females. The ages ranged from 15 to 59 years. The average age, 31½ years.

FISTULA IN ANO, 149 cases; of which only 14 were females, or 1 in about 10½. I do not find it mentioned in surgical works that this disease is most common in males; but the above proportion would seem to indicate that it is so. The disproportion can hardly be accounted for on the natural repugnance of the female sex to submit such lesions to the surgeon's notice; as the cases of hemorrhoids, and other diseases of the rectum requiring operations, in the hospital wards, show no such disproportion between the sexes. Larger tables might change the proportion somewhat, and this will do for a beginning.

FRACTURES.—Fracture of the *ulna* is rare, compared with fracture of the *radius*, and with fracture of both bones, notwithstanding the exposed condition of the olecranon; being in the proportion of 1 to 2. The same holds true in regard to fracture of the fibula, when compared with fracture of the tibia and that of both bones of the leg.

FUNGUS HEMATODES, 17 cases, of which 6 were in males.

HARE-LIP, 86 cases, of which 29 were females.

HIP DISEASE, 181 cases, of which 63 were females.

LUPUS, 8 cases, of which 3 were females.

NERVES, *division of*, for obstinate *neuralgia*, 9 cases.

*Facial*, 3 cases; of which 2 were relieved, 1 not relieved.

*Inferior maxillary*, 1 case, cured.

*Inferior dental*, 1 case, cured.

*Infra-orbital*, 3 cases; 2 cured, 1 much relieved.

*Ulnar*, 1 case, relieved.

STRICTURE OF ŒSOPHAGUS, 14 cases, of which 3 were in females.

TETANUS AND TRISMUS, 8 cases, of which 7 were fatal in from 2 to 19 days.

TORTICOLLIS, 18 cases, of which 5 were in males.

VAGINA, *occlusion of*, 5 cases; operation in 3 cases, of which 1 was successful, and 2 relieved.

VEINS, *introduction of air into*, 2 cases: 1 while removing a tumor in the neck, in which recovery took place; the other into the *axillary* vein, during removal of tumor from the axilla—fatal.

WOUNDS, *suicidal*, *of throat*, 30 cases, of which 4 were females; only 2 cases proved fatal, at the end of five or six days. It is rare that the suicide effects his object in attempting to cut his throat; from ignorance of the situation of the vessels, the cut is generally made too high up and too much in front; by the time the skin is cut, the pain prevents the completion of the suicidal act. The trachea is cut, but the great vessels escape, as we see by the small proportion of deaths among the cases, only 1 in 15.—*Boston Med. and Surg. Journal*, Nov. 27, 1856.

8. PERCHLORIDE OF IRON IN THE TREATMENT OF PANNIFORM KERATITIS.—The medical profession of Lyons, to whom we are in some manner indebted for the introduction of the use of the perchloride of iron as a therapeutical agent, are much interested just now in its application to the treatment of panniform keratitis. This disease is one of great severity, on account of its tenacity, its relapses, and its incessant aggravations, and finally on account of the impairment or total loss of sight to which it leads. Among the numerous methods which surgeons have employed in its treatment, cauterization, and annular division of the vessels supplying the new growth, have doubtless produced successful results; but their efficacy is not such as to leave nothing more to be desired. Their employment is not always easy, and, in the case of infants, oftentimes impossible.

To destroy the very minute vessels running from the surrounding conjunctiva to the surface of the cornea being the principal indication, M. Follin conceived the idea that this might be accomplished by means of the perchloride of iron. This powerful astringent arrested the abnormal circulation by coagulating the blood in the small vessels, which, consequently, being no longer required, were absorbed, and the cornea regained its transparency. Such are the results obtained by MM. Follin, Broca, and Gosselin, in several cases reported.

M. Follin makes use of the perchloride of iron in a perfectly neutral state, at 30° (Beaumé). A single large drop is introduced into the eye by means of a quill. The first effect is a burning pain and a sensation of powerful constriction, which gradually diminish in the course of a quarter of an hour. The heat is, however, more supportable than that produced by many other agents in use, the sulphate of copper for example. If the eye should continue injected and phlogosed, cold applications and gentle astringents should be resorted to; among which latter M. Follin prefers a decoction of rhatany. The perchloride is not repeated for two or three days, and marked amelioration is generally observed after the third or fourth application; the vascularity of the cornea is already diminished, the photophobia has nearly disappeared, and the sight made clear. It is rarely necessary, in order to produce a complete cure, to repeat the remedy oftener than ten or twelve times, frequently four or five applications are sufficient.

The presence of superficial ulcers on the cornea does not contra-indicate the employment of the remedy.—*Archives Générales.*

9. CASE OF SUCCESSFUL LIGATURE OF THE ARTERIA INNOMINATA. By *M. Peixoto.*—Among several interesting "Cases in Surgery" communicated by M. Peixoto to the Academy, we select this one for transcription. M. Moura, a distinguished Portuguese doctor of medicine, aged thirty-three, was the subject of it. An erectile tumor of the right ear began to develop itself in 1832, and in 1845 M. Nelaton tied the posterior auricular, considerable hemorrhage following the fall of the ligature. After temporary amendment, the tumor again made great progress, and frequently gave rise to serious hemorrhage; the patient being then at Rio Janeiro, M. Peixoto tied the common carotid in the middle of its course, 14th November, 1851, and on the 27th surrounded the tumor itself by a ligature, which induced its separation by sloughing. On the 14th of December, some bleeding was observed where the carotid had been tied, the ligature not having yet come away; and as the hemorrhage recurred again, it was resolved to apply a precautionary ligature (*d'attente*) lower down. On the 8th, this was executed on the trunk of the innominata; and in a later communication to the Academy, the author states the cure was completed in two months.

M. Velpeau, reporting on this case,* observes, that, as far as he is aware, this is the first example of a cure resulting from the artificial obliteration of this artery; although the cases of accidental occlusion published by Pelletan, Martin-Solon, and Darrach, had already shown that its occlusion did not deprive either the arm or the brain of a sufficient supply of blood. The cases of operation have hitherto all terminated fatally. Mott, who first practised the operation, in 1818, lost his patient on the twenty-sixth day. Gräfe's patient died on the sixty-eighth day, Bland's on the eighteenth, and Hall's on the sixth. In Lizars' case, death occurred at the end of three weeks. After M. Kuhl's operation, in which the ligature comprised the subclavian and the carotid close to the innominata, death took place on the third day. A patient of M. Arendt's died on the eighth day; and two operations performed by M. Bujalski were followed by death in two or three days. Finally, M. Hutin lost his patient on the eighth day. So that ten operations have furnished as many deaths.

After all, M. Velpeau adds, this is not an example of ligature of the innominata, properly so called; for although a ligature (*d'attente*) was applied to and flattened the vessel, this was not tightened. The patient was cured, but nothing allows us to affirm that the ligature bore rather upon the common trunk than upon the origin of the carotid alone. Nor is there anything that absolutely proves the closure of the innominata, if closed it be, not to have taken place as a consequence of the first ligature, rather than under the mere influence of a ligature *d'attente.—British and Foreign Med. Chir. Review*, Oct. 1856.

10. GLYCERINE AND TANNIN IN VAGINITIS.—In the treatment of this affection, M. Demarquay has found a composition, consisting of eighty parts of glycerine and twenty of tannin, of great service. When the vaginitis first appears, the inflammatory symptoms should be calmed by appropriate regimen, baths, and frequent emollient injections. When the first stage of the inflammation has passed away, and the careful introduction of the speculum has become possible, abundant injections of water are to be thrown in, so as to remove all the muco-pus which lines the walls of the vagina, and these are then dried by a plug of charpie placed at the end of a long forceps. Then, three plugs of wadding, well soaked in glycerine and tannin, are to be introduced. Next day, after a bath, the plugs are removed, new injections made, and the dressing repeated. M. Demarquay has never had to have recourse to more than four or five such dressings. After discontinuing them, astringent injections, consisting of infusion of walnut leaves, in which one drachm of alum to the quart has been dissolved, are employed two or three times a day for a week or ten days.—*Bulletin Thérapeutique,—Ibid.*

* Bull. de l'Acad. tom. xix.

11. CURE OF NAIL IN THE FLESH, WITHOUT OPERATION, BY THE USE OF A SOLUTION OF ACETATE OF LEAD.—The *Correo Medico-Quirurgico* publishes under the above title, a communication to the Surgical Academy of Majorca by Dr. Romualdo Saenz, which contains a clinical verification of the etiological and therapeutical ideas concerning the disease in question, put forth by Professor Van Wangening, of Holland. According to these two observers, the expression *inverted nail*, is incorrect, and leads to improper treatment: the nail does not enter the flesh, but the reverse, the soft parts extending over the nail, in consequence of inflammatory swelling. The indication, therefore, is to cure the chronic inflammation, and repress the fleshy excrescences; a result which may be accomplished by the use of a saturnine wash, as first recommended by Van Wangening.

The diseased parts having been washed with tepid water, the nail should be gently separated from the fungous growth by which it is covered, and two or three drops of the liquid subacetate of lead dropped between them: the parts should then be covered with raw cotton wet with the same liquid. This dressing should be repeated every hour, or every two or three hours, taking care to change the cotton every day, as it becomes hard in the course of twenty-four hours, and will no longer imbibe the solution. The application forms also, upon the surface of the granulations, a solid crust, which it is necessary to remove to prevent purulent accumulation. This dressing should be continued until there is a complete cure.

In proof of the efficacy of this treatment, Doctor Saenz reports several cases in which it was entirely successful. The first was that of a woman, forty years of age, in whom the disease affected the index finger of the right hand: the flesh covered nearly the internal half of the nail, and was the seat of violent pain. Various topical remedies had been employed without avail. Dr. S. ordered the foregoing dressing to be applied, and a cure soon followed.

The second case was that of a woman, who had been a great sufferer for a long time, the disease occupying the ring finger of the left hand. Fungous granulations covered nearly the entire nail. Various unsuccessful applications had been made, including the nitrate of silver. The same treatment was ordered as in the preceding case, and in a short time the disease was entirely cured.

In another case, occurring in the right index finger, the diseased parts had been touched with turpentine, which greatly aggravated the pain. In this, as in the others, the saturnine solution produced a rapid cure.

In many other cases mentioned by Dr. Saenz, including one in which the great toe was affected, the treatment was equally successful.

Finally, Dr. Saenz claims that the acetate fulfils, in the treatment of this disease, all the precepts included in the motto, *cito, tuto, et jucunde*, and is an example of true progress in modern therapeutics, since it forms an effectual and painless substitute for the violent surgical means heretofore employed. The claims of Dr. Saenz are, however, doubtless exaggerated. In many cases in which the inversion depends upon a vicious conformation of the matrix of the nail, and such cases are incontestable, the means of cure are necessarily mechanical, and the solution of acetate of lead comparatively useless; but, on the other hand, we are willing to believe that Drs. Van Wangening and Saenz have cured cases by the means described, and have therefore rendered an essential service to the healing art.—*Revue Thérapeutique Med. Chir.*, Sept. 15, 1856.

12. Spina Bifida.—By *Wm. H. Byford*, of Evansville, Ia.—"Within twelve months past three cases of spina bifida have been brought to my notice, in all of which a peculiarity has been observed which, so far as I know, has not been described by writers upon this subject. The peculiarity has reference to the genital organs mostly; and although in an affection of such general fatality it may not be a matter of much importance, so far as success in management is concerned, yet some interest may attach to it physiologically and pathologically. Two of the cases occurred in my own practice in this city, and the third in the practice of a respectable country practitioner (Dr. Thos. Runcie).

Case I.—Mrs. H. gave birth to her third child, female, June 6th, 1855. It was small, but to all appearances healthy and well formed, with the exception of a spina bifida tumor, occupying the place of the processes of the two lower lumbar vertebræ, about the size of an orange split in half. A circumstance worthy of note, was a tendency in the lower extremities to draw up to the front of the abdomen and chest. They were, when not controlled by interference, extended somewhat rigidly and bent upon the abdomen, so that each foot touched the ear of the side opposite to which it belonged, and it required some force to restore them to their natural position. In about eighteen hours after its birth, the father came to my office and requested me to call, as the mother said something very uncommon was the matter with the child. Upon arriving, she directed my attention to a thick tenacious bloody mucus on the napkin, stating that it came from the vagina, that the child had cried a great deal through the night, and seemed to be affected with bearing-down pains, as shown by its almost constant straining. Upon separating the labia, I found them and the thighs stained by the same discharge as was upon the napkin. The entrance to the vagina was large enough to admit the end of my thumb without obstruction. Protruding between the labia was the neck and body of the tiny uterus, with this mucous discharge issuing from the patulous os tincæ. After a moment's consideration, I oiled my thumb and pressed gently the latter organ upward and backward out of sight. A compress was bound over the closed labia with a T bandage. This afforded so much relief, that the child rested better for several hours; but the descent was repeated, and the organs returned to their places several times before the death of the child. Bearing-down pain invariably returned with the descent, and was relieved by the replacement of the uterus. The child lived about a week, when it died, worn out and exhausted by the suffering and pain, as I thought, from the prolapse. This case, although peculiar in this particular, did not cause any reflection other than a double malformation of any other kind. I supposed it a rare occurrence merely, and not worthy of special note.

Case II.—In less than two months I was called to see another child, delivered the day before, with a spina bifida in the same locality, though not quite so large as the one above-described. I could not learn that the legs assumed the same position as in the first case, but the mother spoke to me of the sanguinolent discharge upon the napkin. The child was female, and upon examination, I found the vaginal opening much larger than natural, and the uterus visible between the labia. It had been crying all night, and straining fearfully. The uterus was made to ascend by very slight pressure upward. This handling did not give any uneasiness, as far as we could judge, and the replacement was followed by complete relief for several hours. This patient lived about ten days, and, as with the other, I thought was exhausted by the pain and watchfulness caused by the prolapse.

"The case of Dr. Runcie (which was the third), was almost exactly like my first. The labia were separated by the prolapsed uterus, the vaginal opening much larger than natural, the same bloody mucus was discharged, and the child was distressed by the same sort of bearing-down pain. In all these cases, the mucus discharged was of that thin tenacious kind furnished by the neck and cavity of the uterus, and in the first instance was seen issuing from the diminutive os tincæ. In a practice of nearly eighteen years, I had not, before last June, seen a case of spina bifida; in the last twelve months I have met with three—all females—all affected with congenital prolapsus uteri. Although unable to say, I apprehend, from the silence of all the authors I can at present consult, that the prolapse is rare. Certainly, however, when it is an accompaniment, it greatly embarrasses the management of the case, and renders the affection more surely fatal. Observing three cases of spina bifida with this peculiarity, occurring close together, induced me to think it might have possibly been hitherto overlooked, and determined me to send them to you for publication. These cases all proved fatal, the first in about one week, the second ten days, and the third three weeks. All of the little patients seemed to suffer a good deal of pain, apparently from the uterine malposition."—*Am. Journal Med. Sci.*, Oct., 1856.

13. TRAUMATIC TETANUS SUCCESSFULLY TREATED BY LOCAL APPLICATION OF CHLOROFORM TO THE SPINE. By *F. Hinkle, M.D.*, of Marietta, Pa.—Mrs. D. E., æt. 38 years, of feeble constitution, the mother of six children, the last being but five months old when the following accident occurred. Having decapitated a large eel, preparatory to stripping off the skin, she attempted to hang the head upon a nail. While doing this, the jaws snapped and caught the index finger of the left hand upon its internal surface, between the second and third phalanges, directly over the joint. The contraction of the jaws was so firm that she was obliged to open them by inserting a knife and forcing them apart.

The pain caused by the bite produced partial syncope. Upon examining the finger she found that the skin was divided in several places along the line made by the jaws. The finger became purple and tumid on its internal surface, attended with acute pain.

She engaged more or less in her household duties from this time until November 16, a period of three weeks, when she consulted me. She informed me that since the accident she had suffered pain of a dull, aching character, at times more or less acute, according as she used the affected hand. She now presented the following symptoms: Pulse 58, very soft and slow; skin hot and dry; tongue slightly coated; bowels had not been moved for two days; acute pain, recurring every fifteen or twenty minutes in the epigastric region, and extending into the hypochondriac region of each side. This affected her breathing, especially when the pain was severe, showing that spasm of the diaphragm was present. Eyebrows contracted; alæ nasi expanded, producing an anxious expression of countenance. It was impossible for her to open her eyes to more than half their extent.

Upon examining the finger, the punctures made by the teeth could be distinctly seen. Pressure over this surface caused acute pain, which darted along the arm, up to her neck, along the spinal column, head and face. There was not a nerve about the head or extremities but what was painful upon pressure. The stiffness, as she termed it, of her face and limbs, with difficult deglutition, had been coming on for four or five days before I was called, she thinking it was only a bad cold. She could not sleep at night on account of spasms, which commenced at the diaphragm and extended to the head and extremities.

*Treatment.*—I directed her to take four compound cathartic pills, to be followed by a dose of castor-oil, if required; and to have her feet bathed in hot water, to which mustard had been added. A large pad of raw cotton covered with muslin to be laid along the whole length of her spine, and half an ounce of chloroform, mixed with one drachm of ether, to be dashed suddenly along the skin; the pad then to be secured over the spine by tapes tied in front of her chest. The patient was now turned on her back, and a drachm of the mixture was applied to the epigastrium, which was also covered with cotton. This afforded almost instant relief to the spasms, and mitigated the pain along the main nerves. The wounded finger was also bathed in chloroform, and well wrapped in cotton. I directed the chloroform to be repeated every two or three hours.

*Nov.* 17. 9 o'clock A.M. No improvement; symptoms the same, but yielded readily to the application of chloroform. When the applications were not made, her sufferings were very much increased. Breathing more labored; bowels moved twice, but not freely. To use the chloroform more frequently, and give the following powder: ℞.—Hyd. chlor. mitis grs. viii; Pulv. ipecac. gr. j.—M. To be followed by a dose of Epsom salts; also to take a spoonful of the following mixture every two or three hours: ℞.—Spts. æther. nit. f ℥j; Ol. terebin. f ʒj; Ext. cannab. ind. ℈j.—M.

18th. 8 o'clock A.M. Found the patient much better. She has had two or three dark-colored dejections. Pulse 60; features less contracted. The pain in finger, cervical ganglia, stiffness of neck and jaw, are all much improved. Can bear more pressure on the spine and along the course of the nerves. To continue the applications of chloroform, particularly to the wounded finger and to the arm and neck of the affected side; and, in addition, to take the following pill every four hours: ℞.—Ext. cannab. ind. gr. j; Mass. hyd. gr. ⅓; Pulv. rhei, grs. iss.—M.

19*th.* Symptoms continue favorable; pulse 60; tongue nearly clean; bowels moved; pain diminished, except in the arm and neck of right side. Countenance still indicative of the disease. Treat. cont.

20*th.* 9½ o'clock A.M. Symptoms improving; pain diminishing. Continue treatment.

21*st.* 11 o'clock A.M. Decidedly better; difficulty of deglutition nearly gone.

22*d.* Feels tolerably well, but says if she could only sleep at nights she would be better; she finds her mind constantly wandering the moment she dozes. Pulse 65; skin moist; tongue clean. She can insert the end of her little finger between her jaws; pains have nearly left her. Treatment continued. Left the patient in good spirits, considering her nearly convalescent.

24*th.* Sent for, in great haste, at 3½ A.M. Found her sinking rapidly; pulse not perceptible at the wrist; features greatly contracted; eyebrows corrugated; jaws closed; eyelids closed, without power to open them; left pupil dilated; skin cool; finger painful; general muscular contraction, especially of the diaphragm; breathing labored and painful; countenance anxious; deep livid hue of face; acute pain every ten minutes, commencing at diaphragm and extending to spinal column, ends of fingers and toes. Could not bear pressure on any part of the body without a jerk, like an electric shock. Gave brandy every fifteen minutes; applied chloroform freely to the epigastrium, spine, neck, arm, and finger. Could not see her tongue; bowels moved yesterday. Gave three ounces of the best brandy with Hoffman's anodyne and tincture of valerian in an hour. Also two grains of ext. cannab. ind. Pulse now gradually rose to 50. Chloroform controlled the pain.

7 A.M. Pulse 54, fuller; countenance more natural and less livid; pupils equal; better temperature of skin; breathing still difficult, but not so painful. I now applied one or two ounces of chloroform every hour or two, just as the pain returned, and thus again succeeded in checking the spasms, which were anticipated the whole day.

1 P.M. Applied caustic iodine to her finger every four hours, so as to blister it freely, but not expose it so much as a fly-blister necessarily would do.

8 P.M. Pulse 55, rather feeble; skin moist and cool; bowels not yet moved; dyspnœa not so constant; otherwise the same as at last visit. Directed her to use brandy and beef-tea freely; gave pills composed of quinine, ext. cannab. ind., and mass. hydrar., every three hours, and to continue the local applications.

*25th.* 10 A.M. Tetanic symptoms milder; longer interval between the pains, and they are less violent; breathing improving; pulse 54, soft and feeble; voice faint; jaws still stiff, as are most of her muscles, yet the chloroform has removed all acute pain. Continue treatment; and, in addition, use twelve drops of the following mixture every two hours: ℞. Spts. æther. nit. f℥ss.; Ol. tiglii, gtt. j.—M. If dyspnœa returns, to use Hoffman's anodyne and tinct. valerian.

A consultation having been agreed upon, Dr. J. Aug. Ehler, of Lancaster City, met me at 7 P.M. We found her slightly improved. Breathing not so painful; skin warmer; countenance more lively and less livid; features still contracted; the other symptoms remained as they were at my last visit. Pulse 54. She has taken half a pint of brandy, with beef-tea. Continued treatment. Dr. E. ordered the cannab. ind. in large doses.

She continued to improve and relapse for four days, supported by brandy, beef-tea, emulsion of carbonate of ammonia and tr. musk.

The bowels were kept regular by castor oil. The ext. cannab. ind. was given to relieve pain and spasm, and the chloroform continued.

*30th.* The disease was now evidently checked, and from this period her recovery gradually took place. By December 10, she was perfectly well.—*Ibid., Oct.*, 1856.

